# Changes in Macrozoobenthos Community after Aquatic Plant Restoration in the Northern Venice Lagoon (IT)

**DOI:** 10.3390/ijerph19084838

**Published:** 2022-04-15

**Authors:** Federica Oselladore, Valentina Bernarello, Federica Cacciatore, Michele Cornello, Rossella Boscolo Brusà, Adriano Sfriso, Andrea Bonometto

**Affiliations:** 1 Italian National Institute for Environmental Protection and Research (ISPRA), Brondolo No. 5, 30015 Chioggia, VE, Italy; federica.oselladore@isprambiente.it (F.O.); valentina.bernarello@isprambiente.it (V.B.); michele.cornello@isprambiente.it (M.C.); rossella.boscolo@isprambiente.it (R.B.B.); andrea.bonometto@isprambiente.it (A.B.); 2 Centre for Estuarine, Coastal and Marine Sciences (CEMAS), Department of Environmental Sciences, Informatics and Statistics, Ca’ Foscari University of Venice, Via Torino 155, 30170 Mestre, VE, Italy; sfrisoad@unive.it

**Keywords:** benthos, transitional waters, ecological indicators, phanerogam transplantation, M-AMBI, LIFE programme

## Abstract

Responses of the macrozoobenthic community to an ecological restoration activity in the northern Venice lagoon were studied, within the scope of the project LIFE SEagrass RESTOration aimed at recreating aquatic phanerogam meadows largely reduced in recent decades. Transplants were successful in almost all project areas. Macrozoobenthos was sampled in eight stations before (2014) and after (2015, 2016, 2017) transplanting activities. An increase in abundance and fluctuations in richness and univariate ecological indices (Shannon’s, Margalef’s, Pielou’s indices) resulted during the years. Comparing non-vegetated and vegetated samples in 2017, every index except Pielou’s increased in the latter. Multivariate analysis (hierarchical cluster analysis, MDS, PERMDISP, SIMPER) grouped samples by localization rather than years, with differences between stations due to the abundance of common species. In 2017, results were also grouped by the presence or absence of aquatic plants, with differences in the abundance of grazer and filter-feeding species. Results of ecological index M-AMBI depicted conditions from moderate to good ecological status (sensu Dir.2000/60/EC) with similar fluctuations, as presented by univariate indices from 2014 to 2017. Responses of the macrozoobenthic community were more evident when comparing vegetated and non-vegetated samples, with the vegetated areas sustaining communities with greater abundance and diversity than non-vegetated samples, thus demonstrating the supporting function of aquatic plants to benthic communities.

## 1. Introduction

Macrozoobenthos comprises all invertebrates, predominantly sedentary and with a long life [1], larger than 1 mm living in close contact with sediment [2]. They play a fundamental role in several environments, especially in ecosystem processes, as they participate in nutrient cycles, metabolization of pollutants, sediment oxygenation, and filtration of phytoplankton [1,3,4]. In addition, they often represent a source of food for organisms placed at higher levels of the food web, such as epibenthic crustaceans, fish, and birds. Thus, they collaborate in the transport of primary production to higher trophic levels [2,3,4].

The main phyla composing soft-bottom macrozoobenthos are polychaetes, molluscs (bivalves and gastropods), and crustaceans (amphipods and decapods) [3].

The distribution of macrozoobenthos depends on both abiotic factors, such as salinity, depth, hydrodynamics, sediment size, and composition, as well as on biotic factors, such as inter- and intraspecific competition and predation [5].

It has also been demonstrated how macrozoobenthos is affected, in a short amount of time, by the presence of anthropogenic pollutants and stressors, even at low concentrations of contaminants [6]. Macrozoobenthos communities are often used as bioindicators to assess the quality of coastal and transitional marine ecosystems [1]. Moreover, they are among the biological quality elements (BQEs) to assess ecological quality in transitional and coastal waters sensu Water Framework Directive (WFD, 2000/60/EC).

Transitional waters (TWs) represent the transition between freshwater and marine environments. Hence, they are characterized by strong gradients and high variability of chemical–physical parameters, such as salinity, ionic composition, temperature, turbidity, dissolved oxygen, pH, redox potential, nutrients, dissolved organic matter, and particulate matter [7]. Coastal lagoons, in particular those near industrialized and/or heavily populated areas, are often characterized by high productivity and poor water quality, due to the accumulation of nutrients, human exploitation of the lagoon’s natural resources, and reduced water exchanges with the sea [8].

TWs, having large fluctuations in environmental variables and accumulation of organic matter, are typically characterized by a reduced diversity of organisms, mainly represented by tolerant species, able to adapt to changes in environmental parameters, often associated with strong dominance of one or a few species [9]. Despite these general features, higher numbers of species and abundance usually characterize TWs with a good environmental status. In this context, the abundance and richness of macrozoobenthos species can be affected also by the presence/absence of aquatic angiosperms [10,11,12,13,14]. Indeed, aquatic angiosperms can increase the complexity of the habitat and provide living spaces and shelter for several species [15,16,17].

One of the most important TWs in the Mediterranean area is the Venice lagoon, placed in the Northern Adriatic Sea and covering an area of about 55,000 ha and with a relatively low average depth of <1 m [8,18,19]. The lagoon is connected to the Adriatic Sea by three inlets: Lido, Malamocco, and Chioggia, from which a network of tidal channels originates and, approaching the mainland, tends to become narrower and shallower, reducing the hydrodynamics in outlying areas [20]. The Burano–Torcello and Malamocco–Marghera canals divide the Venice lagoon into three basins [21]: southern, central, and northern [8].

The southern basin, colonized by wide aquatic angiosperm meadows (*Zostera marina* Linnaeus 1753, *Zostera noltei* Hornemann 1832, *Cymodocea nodosa* (Ucria) Ascherson), is affected by the anthropogenic and port activities that occur at the urban centers of Chioggia and Sottomarina [22].

The central basin is the most directly influenced by human activities, as it receives both wastewaters from Venice, Mestre, and Marghera and the industrial area of Porto Marghera. In the northern basin, the tributaries drain the waters rich in nutrients and fertilizers from an area of intensive agriculture [18]. Algal beds, especially *Ulva rigida*, heavily covered the central and northern basins until the end of the 1990s. Mostly in summer, they increased in coverage and biomass, causing frequent dystrophic crises [12].

In recent decades, aquatic angiosperm meadows have strongly regressed in the Venice lagoon, in particular in the northern area, due to multiple anthropogenic pressures [23], including the high resuspension of sediments due to various activities and the introduction of nutrients and pollutants such as herbicides, widely used in agriculture. However, recent regulatory constraints have greatly reduced the pressure elements, limiting nutrient inputs and regulating clam harvesting [24].

The change in the composition and structure of aquatic vegetation in TWs, due to loss of aquatic angiosperm beds, is one of the main consequences of environmental degradation [25]. In the case of the Venice lagoon, the significant decrease in aquatic angiosperms [23], especially in choked areas, together with the presence of pollutants and nutrients, has affected flora and fauna composition [25]. A reversal of this is possible by adopting restoration measures to recover water quality and significantly reduce nutrients and sediment loads [26]. As a result, aquatic angiosperms would begin to recolonize the lagoon [23,27,28,29].

In this context, the project LIFE SERESTO “Habitat 1150* (Coastal lagoon) recovery by SEagrass RESTOration” (LIFE12 NAT/IT/000331, www.lifeseresto.eu, accessed on 13 April 2022) was carried out from 2014 to 2017. Its main objective was the restoration and conservation of the 1150* priority aquatic habitat (coastal lagoons) in the SIC Laguna Superiore di Venezia (IT3250031), covering approximately 3660 ha, through transplants of submerged aquatic angiosperms, especially *Z. marina* and *Z. noltei*. Other objectives of the project were to improve the ecological status of TW bodies (sensu WFD) and to improve and preserve the state of coastal lagoon habitats (sensu Dir. 92/43/EEC, Habitat Directive) and associated ecosystem services [30].

The research presented in this paper was part of the LIFE SERESTO project, and its purpose was to study the response of the macrozoobenthic community following ecological restoration interventions of aquatic angiosperm transplantations. Comparisons between ante and post operam results, as well as vegetated and non-vegetated areas, confirmed the usefulness of macrozoobenthic communities as a sensitive bioindicator in restoration projects in transitional waters.

An improvement in the macrozoobenthonic community over time was expected, both as regards the richness of species and as regards the number of individuals. It was further expected that aquatic angiosperm transplants would improve the macrozoobenthos community in terms of richness and abundance in the transplanted areas.

## 2. Materials and Methods

### 2.1. Study Area and Sampling Site

**Figure 1:**
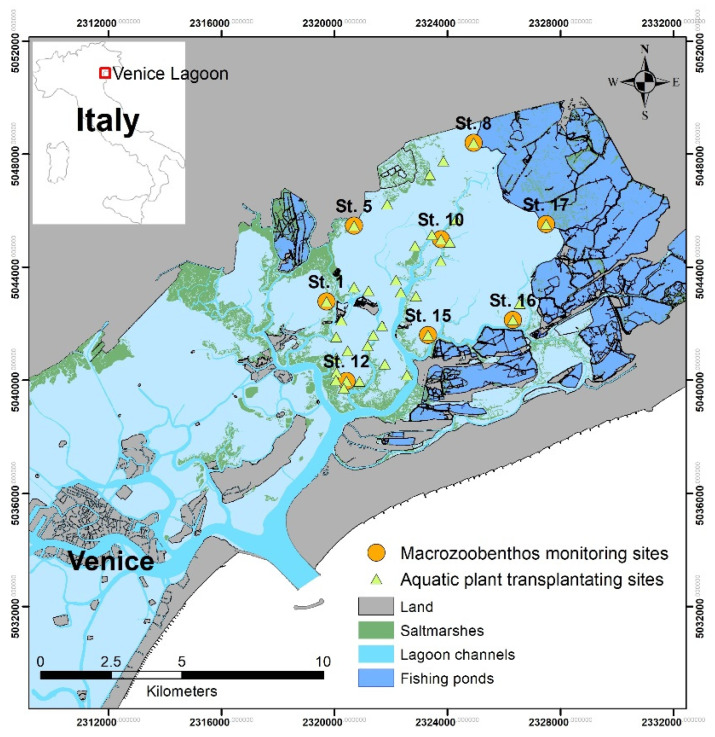
Map of the sampling area.

This study was conducted in an area (36.6 km^2^) of the northern lagoon of Venice in the SIC IT3250031 Laguna Superiore di Venezia (Figure 1), a typical tidal lagoon environment characterized by the presence of a complex system of sandbanks, canals, and saltmarshes.

According to the Directive 2000/60, the study area belongs to two water bodies (WsB): the polyhaline PC1-Dese and the euhaline EC-Palude Maggiore [31]. Macrozoobenthos was sampled in eight stations during the spring season before (2014) and after (2015, 2016, 2017) transplanting activities. In 2017, samples were collected both on bare sediments (NVS), and on those planted (VS), in order to highlight the differences and benefits of aquatic plant restoration.

### 2.2. Sampling Procedure and Sample Treatment

Samples were collected with an Ekman–Birge grab (sampling area: 0.0225 m^2^), able to penetrate the sediment up to approximately 25 cm [2]; for each site, three subsamples were taken.

Each sample was sieved with a 1 mm mesh sieve. The material was immersed in an anesthetizing solution of magnesium chloride (MgCl_2_) [2]. The samples were then collected, labeled, and kept refrigerated at 4–6 °C until arrival in the laboratory where they were frozen at −20 °C [12].

In the laboratory, the samples were washed and sieved with a 0.5 mm mesh sieve and sorted [2]. The organisms were then classified down to the lowest possible taxonomic level [2,12]. The taxa have been named according to the World Register of Marine Species (WoRMS, http://www.marinespecies.org/, accessed on 24 January 2022).

### 2.3. Data Analysis

Macrozoobenthos was analyzed using univariate and multivariate techniques and multivariate ecological indices. The metrics considered include abundance, richness [2], and diversity indices such as Shannon’s, Margalef’s, and Pielou’s [12].

Abundance data (transformed with log (x + 1) function) were used to create a similarity matrix based on the Bray–Curtis similarity index. The matrices were used to perform hierarchical cluster analysis (CLUSTER) and nonmetric multidimensional scaling ordination (MDS) [12,32,33], in order to evaluate similarities between samples (grouped by stations, sampling years, and type of seabed-transplanted vs. bare). To evaluate the contribution of the species that determined the differences between the groups created by the cluster analysis, the similarity percentages were calculated (SIMPER analysis) [32]. PERMDISP analysis was used to calculate an F-statistic, in order to assess whether the dispersions between groups defined with MDS and CLUSTER analyses were significant.

Univariate and multivariate analyses were performed using the PRIMER-E v.6.1 software package [34] with PERMANOVA+ and STATISTICA v.6 [35].

Starting from richness and abundance data, the biotic indices multivariate marine biotic index (M-AMBI) [36] were also calculated to evaluate the conditions of the benthonic community of the study area [37] using the software AZTI (version 5.0) with the species list version of May 2014.

## 3. Results

### 3.1. Univariate Analysis

A total of 5860 individuals were sampled and identified between 2014 and 2017, belonging to 115 taxa: 93 at the species level, 11 at the genus level, and 11 at the family level. The complete list of *taxa* is reported in Table S1.

**Figure 2:**
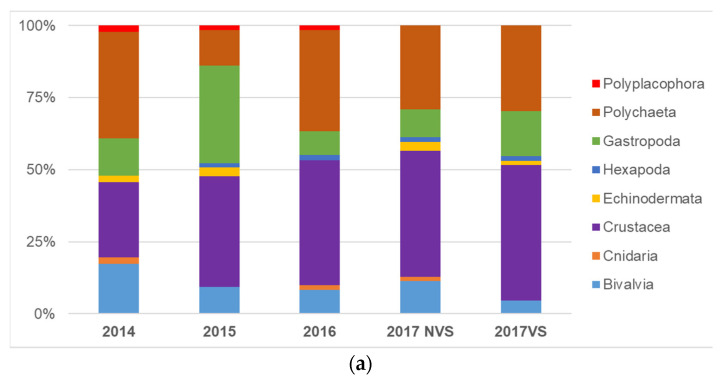

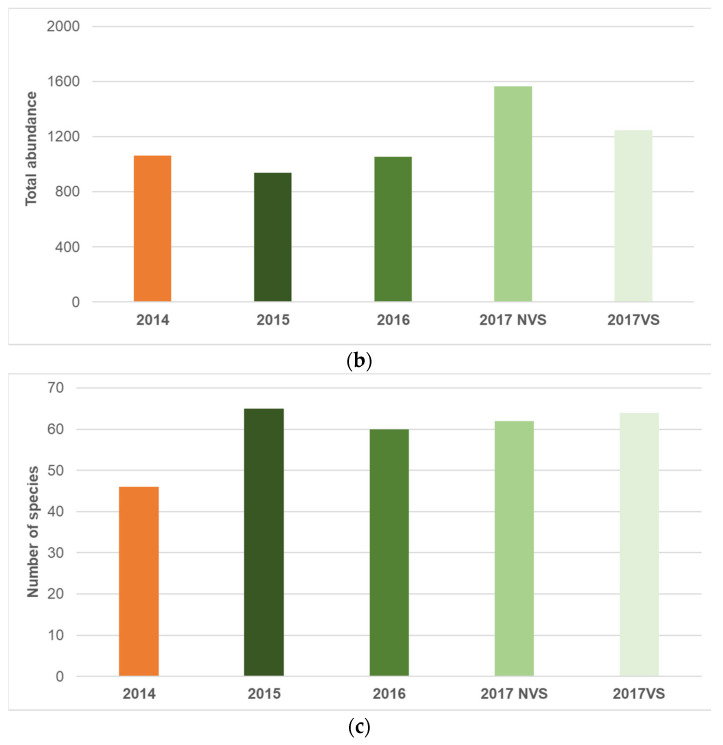
(**a**) Distribution of collected species in the most represented taxa; (**b**) total abundance and (**c**) number of species from 2014 ante transplant operations to 2017 post-transplant operations (vegetated –VS- and non-vegetated stations –NVS-).

Crustaceans were the most represented group during the whole period, both in terms of abundance and number of species. They made up to 40% of the total, mainly determined by the amphipods species *Caprella mitis* Mayer, 1890 and *Gammarus insensibilis* Stock, 1966. They were followed by polychaetes (28%) mainly characterized by *Aphelochaeta multibranchis* (Grube, 1863) and *Nephtys hombergii* Savigny in Lamarck, 1818, gastropods (17%) mainly with *Bittium reticulatum* (da Costa, 1788) and bivalves (10%), mainly represented by the species *Abra segmentum* (Récluz, 1843) and *Cerastoderma glaucum* (Bruguière, 1789). Lower percentages (1–2% of the total) were recorded for hexapods (larvae), echinoderms, cnidarians, and polyplacophorans (Figure 2a).

Total abundance (Figure 2b) has increased over the years from 1064 individuals sampled before transplant operations (2014) to 1563 found after the end of transplants in 2017 at non-vegetated stations (NVS) and 1245 at vegetated stations (VS). The number of species (Figure 2c) remained almost constant in the post-transplant operation monitoring years (65 in 2015, 60 in 2016, 62 in NVS, and 64 in VS in 2017) but increased compared to 2014, when only 46 species were found.

**Figure 3:**
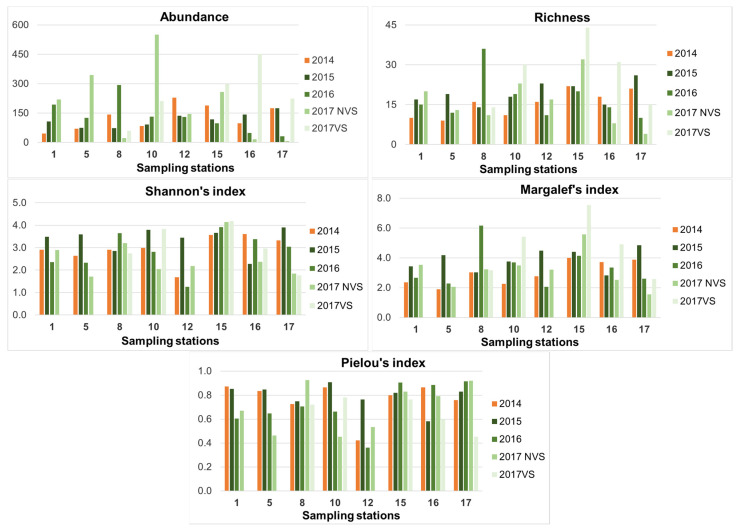
Ecological indices at each station from 2014 ante transplant operations to 2017 post-transplant operations (vegetated –VS- and non-vegetated stations –NVS-).

The ecological indices (Figure 3) showed fluctuations over the years, but they were usually higher at VS in 2017, with the exception of Pielou’s index.

Abundance per station varied from a minimum of 7 at St.17 NVS in 2017 to a maximum of 551 at the St.10 VS in 2017. Considering the mean values, the lowest values were recorded in 2015 (114.4 ± 35.2) and the highest in 2017 at VS (249.0 ± 142.6).

The richness showed the lowest values again at St.17 NVS in 2017 (4 species), while the highest values were recorded at st.15 VS in 2017 (44 species). Considering the mean value of richness for all stations, the lowest values resulted before transplants in 2014 (15.4 ± 4.9) and the highest in VS in 2017 (26.8 ± 12.6).

Shannon’s index mean values were higher in the post-transplanting monitoring years 2015 and 2017 at VS (3.4 ± 0.5 and 3.1 ± 0.9, respectively), with the maximum values reached at St. 15 in 2017 (VS and NVS: 4.2 and 4.1, respectively) and the lowest value at St.12 in 2016 (both VS and NVS 1.2). The Margalef’s index mean values were higher at VS in 2017 (4.7 ± 2.0), with the maximum value at St.15 in 2017 (7.5) and the minimum value at St.17NVS in the same year (1.5).

Pielou’s index, on the contrary, showed an inverse trend, with higher average values detected in ante transplantations in 2014, early post-transplant operations in 2015 (both 0.8 ± 0.1), and slightly lower mean values in the other surveys (0.7 ± 0.2 in 2016 at all stations and in 2017 at NVS; 0.7 ± 0.1 in 2017 at VS).

Application of Kruskal–Wallis tests revealed, however, that there were no significant differences in any of the indices described above, regarding comparisons between years and stations (both vegetated and non-vegetated); therefore, the comparisons remain only qualitative.

### 3.2. Multivariate Analysis

Multivariate analyses showed clusters linked to the sampling period (ante vs. post-transplant operations) and similar environmental characteristics, including the success of transplants in various stations.

**Figure 4:**
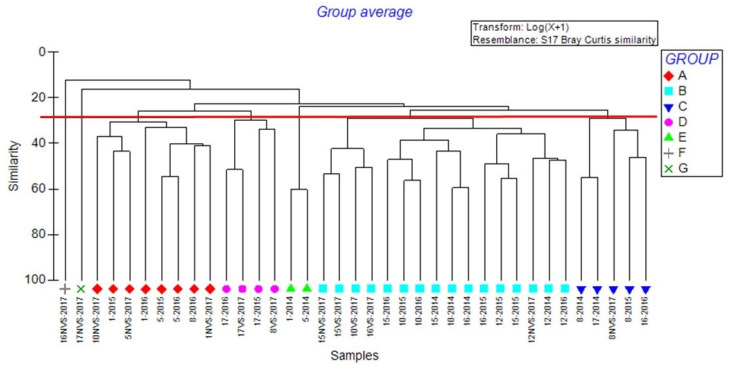
Cluster analysis applied to the abundance data at the eight stations sampled from 2014 ante transplantation to 2017 post-transplantation (vegetated –VS- and non-vegetated stations –NVS).

Cluster analysis (Figure 4) shows the presence of four main groups, at a similarity level of 28%: Group A, with Stations 1 and 5 with the exception of 2014; Group B, with the majority of Stations 10, 12, 15, and 16; Group C, with Station 8 except 2016; and Group D, with Station 17 with the exception of 2014. Groups were statistically significant applying the PERMDISP test (F = 27.132; *p* = 0.001). In the pairwise comparison, Groups C and D were not significantly different, and they both contained the majority of Stations 8 and 17. In addition, the comparison between Group E with Groups A and B was not statistically significant. Group A comprised only Stations 1 and 5 of 2014, and Group A contained all other Stations 1 and 5. Generally, groups indicated both spatial difference and difference between before and after transplants.

**Figure 5:**
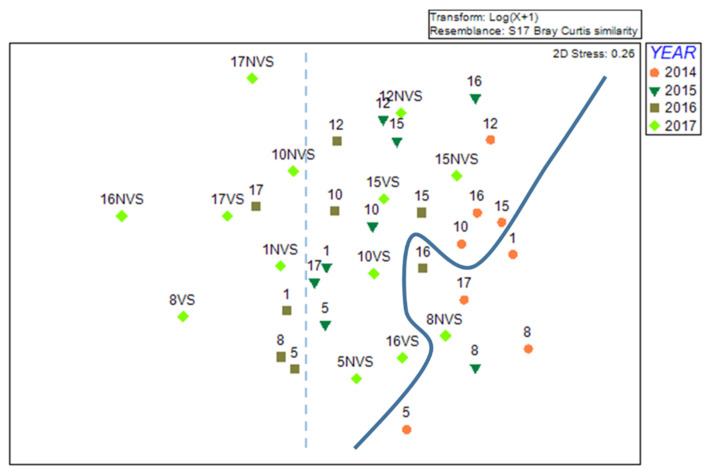
MDS plot applied to the abundance data of the eight samples grouped by year.

In the MDS plot (Figure 5), the projection of samples of a multidimensional space is shown. On the left of the dotted line, the samples forming Groups A, D, F, and G in the cluster analysis are placed. On the right of the solid line, there are the samples of Groups C and E. Samples of Group B are placed between the two lines. In the plot, the 2014 samples are separated from the others (on the right side).

SIMPER analysis indicates a dissimilarity percentage between 74.3 and 91.0% between cluster analysis groups. Differences were determined mainly by higher or lower abundance of some of the most common species, such as *G. insensibilis*, *C. mitis*, *B. reticulatum*, *A. segmentum*, and *N. hombergii*.

**Figure 6:**
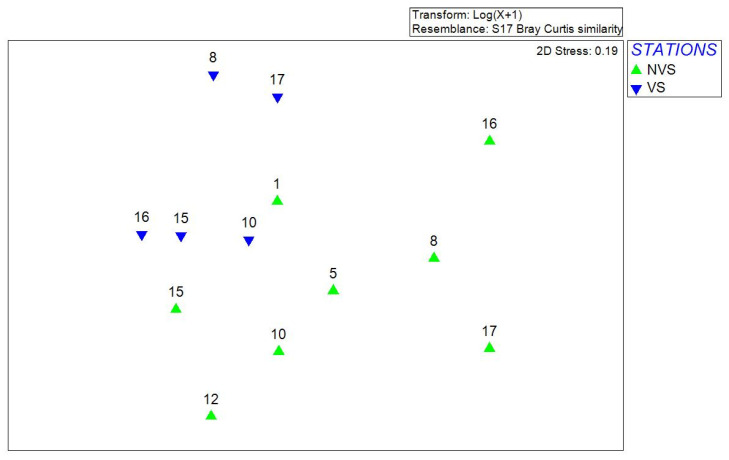
MDS plot applied to only 2017 samples gathered on vegetated (VS) or non-vegetated (NVS) sediments.

The MDS plot in Figure 6 shows the separation between vegetated (VS) stations and non-vegetated (NVS) stations sampled in 2017. The distance between the two groups is statistically different applying the PERMDISP test: F = 7.6567; *p* = 0.031.

SIMPER analysis showed a percentage of dissimilarity of 77.64% between vegetated and non-vegetated stations. The greater abundance of the gastropods *Steromphala adriatica* (Philippi, 1844) and *B. reticulatum* at VS mainly determined dissimilarity results.

### 3.3. Ecological Index

M-AMBI values (Table 1) showed fluctuations over the years, indicating a general improvement in the study area, especially at the end of post operam monitoring in VS (2017 VS).

## 4. Discussion

The monitoring of the macrozoobenthos community in the northern Venice lagoon before and after restoration actions of the LIFE SERESTO project showed some changes due to the transplantation of aquatic angiosperms.

The distribution of organisms (Crustaceans > Polychaetes > Gastropods > Bivalves) reported in this study is typical of the Venice lagoon [2,12,38] and generally of Adriatic lagoons located in the Po River Delta [39], Apulia Region-Lesina [40].

Comparison between 2014 (ante operam) and the following years showed that in the post operam period, there was an increase in the total macrozoobenthos abundance and, above all, in the total number of species, passing from an initial value of 46 in 2014 to 64 in 2017 in the vegetated areas.

A significant increase in the number of species and abundance of macrozoobenthos was also reported in several aquatic angiosperm transplantation experiments, such as in the southern Venice lagoon [41]; in the Northern Adriatic Sea [42]; in Indonesia [43,44]; in North Carolina, U.S. [45]; in Florida, U.S. [46]; and in Oregon, U.S. [47].

Furthermore, studies specifically focusing on seagrass transplantation showed that, in transplanted areas, richness and abundance of benthic fauna tend to be higher than in bare sediments [12,42,44,45,47].

Moreover, lagoon areas and shallow bays with aquatic angiosperms showed higher values in abundance, biomass, and species richness when compared to areas characterized by macroalgae or bare sediments [12,45,47,48,49]. The presence of submerged phanerogams can promote the colonization process of new benthic species thanks to the increase in spatial heterogeneity created by the settlement of the plants [12,42,44,45,46,47,50,51]. The aquatic plant leaves also help to reduce water turbulence [47,52,53], favoring the deposit of fine sediment enriched with organic fraction, which is a source of nutrients for all filter feeders [12,43,47].

Finally, aquatic angiosperms contribute to improving the oxygenation conditions of surface sediments [12,43] and implement a shading action on the benthonic community, which is particularly important in the summer season [54,55].

In addition, in the project areas, there was also an increase in fish fauna in the first year after the start of the transplant operations, probably due to not only an increase in the complexity of the substrate, but also an increase in the availability of prey, such as macrozoobenthos species [56].

Density values, registered in 2002 in the same area of this study, varied from 22 to 430 ind/m^2^ [57], with a distribution defined, above all, by the degree of confinement of the sampling stations [38,58]. Between 2014 and 2017, instead, the density of macrozoobenthos organisms was higher, varying from 1733 ind/m^2^ in 2014 to 2600 ind/m^2^ in 2017, with values more similar to those sampled in the eaves area of the Dese river [57,58]. Lower density values recorded in 2002 can also be attributed to the fact that, as reported by Sfriso et al. [56] and Rismondo et al. [28], in the early 2000s, submerged aquatic angiosperms disappeared almost completely in the northern basin of the Venice lagoon.

Studies conducted in North Carolina also showed that the density of benthic individuals was higher in vegetated sites rather than in bare sediments [45]; in Indonesia, on the other hand, the density increased over time after seagrass transplant operations [43].

The analysis of the main ecological indices showed a substantial improvement in the conditions of the sampling area in the post operam period, especially in those sites where aquatic angiosperm transplantation was successful, although fluctuations over the years and in the sampling stations were evident. This was also reported in the previously cited studies carried out in different areas [53,55] and in other studies conducted in the United Kingdom and China [59,60].

The Shannon’s index results, which on average had a higher value in the post operam and vegetated sites, also showed values higher than those carried out in a completely comparable area in 1991 [32,33].

Station 15, less confined and near a canal that directly connects the lagoon and the sea, showed the best values of the index. In the lagoon areas where exchanges with the sea are greater, there is a tendency to have benthic communities with higher richness, since even the most euryhaline species have the ability to settle in these areas [32,57].

Margalef’s index in 2002 showed values between 1 and 3 in the same study area [57], while in this study, the values found were higher, ranging between 2.9 in 2014 and 4.7 in 2017, more similar to those usually found in less confined areas [38,57].

Pielou’s index values showed instead an inverse trend, with higher values in 2014 and lower average values in the following years, even if the maximum values were recorded in the post operam period, demonstrating greater heterogeneity within the benthic community. This is explained by the fact that starting from 2015, there was an increase in the abundance of amphipods, in particular of species *G. insensibilis*, *Gammarus aequicauda* (Martynov, 1931), *Erichthonius brasiliensis* (Dana, 1853), and Caprellidae.

The two species of *Gammarus*, which are often used as indicators of the quality of the environment [61,62], had an increase from 2015. *G. insensibilis*, in particular, showed a greater increase in the number of individuals. It is a species sensitive to the changes in the hydromorphological conditions of the environment and a good indicator of disturbed environmental conditions [62,63,64]. Its increase in the studied sites, therefore, could be correlated with an improvement in the hydromorphological conditions.

All the species mentioned are, at least partially, suspension feeders [61]. Their increase could be due to the fact that the presence of aquatic angiosperms favored the persistence and deposition of fine sediments in the study area [43,47,52,53].

The data ordination performed with the MDS technique has clearly shown that the sampling of 2014 forms a separate group compared to the post operam samples. This confirms what has already emerged through the univariate analysis and the study of ecological indices, and was further statistically validated by the PERMDISP test, which showed significant differences in the comparison between 2014 and all the subsequent years.

Therefore, the multivariate analysis also underlines the importance of aquatic angiosperms in environments characterized by a high degree of heterogeneity such as coastal lagoons, and, above all, the need to implement new environmental restoration plans aimed at improving and restoring the lagoon environment.

The multivariate analysis also highlighted the formation of groups between stations with similar characteristics, such as Stations 1 and 5, characterized by greater eutrophication and less success of transplants; Stations 10, 12, 15, and 16, with intermediate conditions; and Stations 8 and 17, characterized by low nutrients, low sedimentation rates, and greater transplant success.

Through SIMPER analysis, it was then possible to identify the species that characterize the differences between the groups of stations that emerged in the multivariate analysis. This difference was mainly given by five species, which may have a greater or lesser abundance in each group: *G. insensibilis*, *C. mitis*, *B. reticulatum*, *A. segmentum*, and *N. hombergii*. The two amphipods species, as already mentioned above, are very sensitive to environmental conditions [61], and probably even on a small scale, the difference in the chemical–physical parameters of the stations can determine a greater or lesser abundance of the species. The polychaete *N. hombergii* has typically carnivorous habits [65,66]. It is more abundant in those stations in which there are simultaneous increases in the number of potential prey, such as crustaceans [67,68]. The abundance of the bivalve *A. segmentum* in Stations 1 and 5 is in line with what was stated in a previous study carried out in the same area where the two stations are located [55]. On the contrary, the gastropod *B. reticulatum* was found in the stations where the transplant operations were successful. This species is indeed a grazer, and its feeding habit is favored by the presence of aquatic angiosperms [12,55,58,65,66].

A comparison was then made for 2017 only between the benthic populations sampled in the vegetated stations and those in the bare sediments, but it had no significant results. However, the grazer *S. adriatica* and *B. reticulatum* determined differences between stations. They are usually very abundant in vegetated areas, where they can find greater sources of nutrition [69,70].

To evaluate the ecological status conditions of the benthonic community of the study area, M-AMBI [36] was also applied with reference to boundaries set by Italian regulations (Environmental Ministry Decree 260/2010) implemented by the European Water Framework Directive (WFD) 2000/60/EC.

In this work, the index showed fluctuations over the years, although there was generally an increase in the average values of the index compared to 2014 [25], due to a consequent improvement in the conditions of the benthic community, even more evident in the stations vegetated by aquatic angiosperms.

However, it should be emphasized that the benthic community is also characteristic of the fact that it responds slowly to both positive and negative pressures [2,42].

The general improvement in the study area observed so far was most likely linked to transplantations of phanerogams.

## 5. Conclusions

Results of the present study, carried out within the LIFE SERESTO project, showed changes in the macrozoobenthic community, after aquatic angiosperm transplantation activities in the northern Venice lagoon to recover the aquatic angiosperms habitat.

Indeed, there was a differentiation between the populations sampled in 2014 (ante operam) compared to subsequent years (post operam: 2015–2017).

Differences between the populations after transplanting were also evident, mainly due to the hydromophrological differences between the stations themselves. Transplants, indeed, led to an increase in spatial heterogeneity, a typical characteristic of TW environments, with a consequent increase in the diversity of habitats available to the various macrozoobenthic populations.

Data confirmed the importance of aquatic angiosperms for the structuring of the macrozoobenthic community, especially evident in the comparison between vegetated and non-vegetated stations.

Further studies will allow assessing the long-term effects of phanerogam transplant operations on the macrozoobenthic community, given the slower response speed of the organisms.

## Figures and Tables

**Table 1:** M-AMBI results from 2014 ante transplant operations to 2017 post-transplant operations.

	Years				
Stations	2014	2015	2016	2017 NVS	2017 VS
1	0.73	0.91	0.7	0.91	
5	0.69	0.96	0.65	0.58	
8	0.53	0.51	0.8	0.59	0.59
10	0.54	0.68	0.66	0.6	0.8
12	0.68	0.95	0.61	0.79	
15	0.59	0.69	0.68	0.76	0.88
16	0.62	0.46	0.55	0.44	0.78
17	0.67	0.76	0.55	0.49	0.59

(NVS = non-vegetated; VS = vegetated. The colors indicate the ecological classifications: green = good; yellow = moderate; orange = poor; red = bad).

## Data Availability

The data presented in this study are available on request from the corresponding author. The data are not publicly available.

## Supplementary Materials

The following supporting information can be downloaded at: https://www.mdpi.com/article/10.3390/ijerph19084838/s1, Table S1: List of determined taxa from 2014 to 2017.
