# Supplementary material for: Changes in Macrozoobenthos Community after Aquatic Plant Restoration in the Northern Venice Lagoon (IT)
Source: Int J Environ Res Public Health. 2022 Apr 15;19(8):4838. doi: 10.3390/ijerph19084838 (PMC9029909; doi:10.3390/ijerph19084838)
Supplement: Supplementary file 1 [file ijerph-19-04838-s001.zip › ijerph-1629558-supplementary.pdf]

Supplementary Materials

Table S1. List of determined taxa from 2014 to 2017.

Phylum	Class	Order	Family	Taxa	Author
Anellida	Polychaeta	Capitellida	Capitellidae	*Capitella capitata*	(Fabricius, 1780)
				Heteromastus filiformis	(Claparède, 1864)
				Mediomastus capensis	Day, 1961
				Notomastus lineatus	Claparède, 1869
				Notomastus sp.	
				Pseudoleiocapitella fauveli	Harmelin, 1964
				Capitellidae undet.	
		Eunicida	Eunicidae	*Marphysa sanguinea*	(Montagu, 1813)
				Eunicidae undet.	
		Phyllodocida	Aphroditidae	*Aphrodita aculeata*	Linnaeus, 1758
			Glyceridae	*Glycera tridactyla*	Schmarda, 1861
				Glycera sp.	
			Phyllodocidae	*Mysta picta*	(Quatrefages, 1866)
				Phyllodoce schmardaei	Day, 1963
				Phyllodoce sp.	
			Nephtyidae	*Nephtys cirrosa*	Ehlers, 1868
				Nephtys hombergii	Savigny in Lamarck, 1818
				Nephtydae undet.	
			Nereididae	*Alitta succinea*	(Leuckart, 1847)
				Hediste diversicolor	(O.F. Müller, 1776)
				Neanthes acuminata	(Ehlers, 1868
				Perinereis cultrifera	(Grube, 1840)
				Platynereis dumerilii	(Audouin & Milne Edwards, 1833
				Nereididae undet.	
			Syllidae	Syllidae undet.	

		Sabellida	Serpulidae	*Hydroides dianthus*	(Verril, 1873)
		Terebellida	Cirratulidae	*Aphelochaeta multibranchis*	(Grube, 1863)
				Chaetozone setosa	Malmgren, 1867
				Cirratulus cirratus	(O.F. Müller, 1776)
				Cirriformia tentaculata	(Montagu, 1808)
				Tharyx sp.	
				Cirratulidae undet.	
			Ampharetidae	*Melinna palmata*	Grube, 1870
			Maldanidae	*Euclymene oerstedi*	(Claparède, 1863)
				Leiochone leiopygos	(Grube, 1860)
				Maldanidae undet.	
		Spionida	Spionidae	Spionidae undet.	
Arthropoda	Insecta	Diptera	Chironomidae	*Chironomus salinarius*	Kieffer, 1915
	Malacostraca	Amphipoda	Ampeliscidae	*Ampelisca sarsi*	Chevreux, 1888
				Ampelisca sp.	
			Amphitoidae	*Pleonexes helleri*	(Karaman, 1975)
			Aoridae	*Microdeutopus gryllotalpa*	Costa, 1853
				Microdeutopus anomalus	(Rathke, 1843)
				Microdeutopus sp.	
				Microdeutopus versiculatus	(Spence Bate, 1857)
			Caprellidae	*Caprella acanthifera*	Leach, 1814
				Caprella equilibra	Say, 1818
				Caprella mitis	Mayer, 1890
				Caprella scaura	Templeton, 1836
				Caprella sp.	
				Caprellidae undet.	
				Pseudolirius kroyeri	(Haller, 1879)
			Corophiidae	*Corophium orientale*	Schellenberg, 1928
				Corophium sp.	

		Monocorophium insidiosum	(Crawford, 1937)
	Dexaminidae	*Dexamine spinosa*	(Montagu, 1813)
	Gammaridae	*Gammarus aequicauda*	(Martynov, 1931)
		Gammarus insensibilis	Stock, 1966
		Gammaridae undet.	
	Ischyroceridae	*Ericthonius brasiliensis*	(Dana, 1853)
		Ericthonius punctatus	(Spence Bate, 1857)
		Jassa cfr *cadetta*	Krapp, Rampin & Libertini, 2008
	Leucothoidae	*Leucothoe oboa*	Karaman, 1971
		Leucothoe incisa	Robertson, 1892
	Maeridae	*Elasmopus* sp.	
		Maera grossimana	(Montagu, 1808)
		Maera sp.	
	Melitidae	*Melita palmata*	(Montagu, 1804)
	Nuuanuidae	*Gammarella fucicola*	(Leach, 1814)
	Oedicerotidae	*Perioculodes aequimanus*	(Kossman, 1880)
		Deflexilodes subnudus	(Norman, 1889)
	Stenothoidae	*Stenothoe monoculoides*	(Montagu, 1813)
Cumacea	Bodotriidae	*Iphinoe adriatica*	Băcescu, 1988
Decapoda	Carcinidae	*Carcinus aestuarii*	Nardo, 1847
	Palaemonidae	*Palaemon adspersus*	Rathke, 1836
	Processidae	*Processa modica modica*	Williamson in Williamson & Rochanaburanonm, 1979
	Upogebiidae	*Upogebia pusilla*	(Petagna, 1792)
		Upogebia tipica	(Nardo, 1869)
Isopoda	Anthuridae	*Cyathura carinata*	Krøyer, 1847
	Idoteidae	*Idotea chelipes*	(Pallas, 1766)
		Idotea balthica	(Pallas, 1772)
Lepadiformes	Lepadidae	*Lepas* sp.	

		Tanaidacea	Apseudidae	*Apseudopsis latreillii*	(Milne Edwards, 1828)
			Leptocheliidae	*Chondrochelia savignyi*	(Kroyer, 1842)
			Tanaididae	*Tanais dulongii*	(Audouin, 1826)
Cnidaria	Anthozoa	Actiniaria		Actiniaria undet.	
Echinodermata	Ophiuroidea	Amphilepidida	Ophiotrichidae	*Ophiothrix fragilis*	(Abildgaard in O.F. Müller, 1789)
		Dendrochirotida	Cucumariidae	*Paraleptopentacta elongata*	(Düben & Koren, 1846)
Mollusca	Bivalvia	Cardiida	Cardiidae	*Cerastoderma glaucum*	(Bruguière, 1789)
				Papillicardium papillosum	(Poli, 1791)
			Semelidae	*Abra alba*	(W. Wood, 1802)
				Abra prismatica	(Montagu, 1808)
				Abra segmentum	(Récluz, 1843)
				Scrobicularia plana	(da Costa, 1779)
		Galeommatida	Lasaeidae	*Hemilepton nitidum*	(W. Turton, 1822)
		Lucinida	Lucinidae	*Loripes orbiculatus*	Poli, 1795
				Loripinus fragilis	(Philippi, 1836)
		Mytilida	Mytiliae	*Mytilus galloprovincialis*	Lamarck, 1819
				Musculus subpictus	(Cantraine, 1835)
		Nuculida	Nuculidae	*Nucula nucleus*	(Linnaeus, 1758)
		Venerida	Veneridae	*Polititapes aureus*	(Gmelin, 1791)
				Ruditapes philippinarum	(Adams & Reeve, 1850)
	Gastropoda	Archeogastropoda	Trochidae	*Steromphala adriatica*	(Philippi, 1844)
		Caenogastropoda	Cerithiidae	*Bittium reticulatum*	(da Costa, 1778)
		Cephalaspidea	Haminoeidae	*Haminoea navicula*	(da Costa, 1778)
		Littorinomorpha	Hydrobiidae	*Ecrobia ventrosa*	(Montagnu, 1803)
			Rissoidae	*Pusillina lineolata*	(Michaud, 1830)
				Pusillina sarsii	(Lovén, 1846)
				Rissoa splendida	Eichwald, 1830
		Neogastropoda	Nassariidae	*Tritia neritea*	(Linnaeus, 1758)

			Tritia nitida	(Jeffreys, 1867)
	Trochida	Phasianellidae	*Tricolia pullus*	(Linnaeus, 1758)
		Trochidae	*Steromphala varia*	(Linnaeus, 1758)
		Phyramidellidae	*Odostomia* cfr *nardoi*	Brusina, 1869
Polyplacophora	Chitonida	Chitonidae	*Rhyssoplax olivacea*	Spengler, 1797
